# Editorial Comment: Sacral neuromodulation versus onabotulinumtoxinA for refractory urgency urinary incontinence: impact on fecal incontinence symptoms and sexual function

**DOI:** 10.1590/S1677-5538.IBJU.2020.04.13

**Published:** 2020-05-20

**Authors:** Cássio L. Z. Riccetto

**Affiliations:** 1 Divisão de Urologia Feminina Faculdade de Ciências Médicas Universidade Estadual de Campinas CampinasSP Brasil Divisão de Urologia Feminina - Faculdade de Ciências Médicas da Universidade Estadual de Campinas – UNICAMP, Campinas, SP, Brasil

Andy UU ^1^, Amundsen CL ^2^, Honeycutt E ^3^, Markland AD ^4^, Dunivan G ^5^, Dyer KY ^6^, et al.

^1^ Department of Obstetrics and Gynecology, University of Pennsylvania, Philadelphia, Pennsylvania; ^2^ Department of Obstetrics and Gynecology, Duke University Medical Center, Durham, North Carolina; ^3^ RTI International, Research Triangle Park, North Carolina; ^4^ Department of Medicine, University of Alabama at Birmingham, Birmingham, Alabama; 5 Departments of Obstetrics and Gynecology and Surgery, University of New Mexico Health Sciences Center, Albuquerque, New Mexico; ^6^ Department of Obstetrics and Gynecology Kaiser Permanente, San Diego, California

Am J Obstet Gynecol. 2019 Nov;221(5):513.e1-513.e15

**DOI: 10.1016/j.ajog.2019.06.018 | ACCESS: 10.1016/j.ajog.2019.06.018**

## COMMENT

The prevalence of double incontinence among Brazilian women is 4.9% and its incidence in the period between 2006 and 2010 was 13.8/1000 person/year ( 1 ) In fact, evacuatory and even sexual symptoms are usually underestimated in urological consultations related to overactive bladder (OAB) symptoms. In those women with refractory OAB, the coexistence of these dysfunctions may be even greater. The effects of sacral neuromodulation (SNM) on fecal incontinence are well known, so that this treatment represents an important therapeutic option in double incontinent patients. On the other hand, the therapeutic mechanism of intravesical injection of botulinum toxin (BTX) is much less understood. In this prospective randomized study, the authors performed a post-hoc analysis of data from the ROSETTA trial ( 2 ), which included women with refractory urinary incontinence treated with BTX-A (n: 190) or SNM (n: 174). Urinary incontinence and sexual symptoms were evaluated for up to 24 months using the Pelvic Organ Prolapse / Urinary Sexual Incontinence Questionnaire -12 (PISQ-12), IUGA -Revised (PISQ-IR) and St Mark’s (Vaizey) Fecal Incontinence Severity Scale. The incidence of fecal incontinence (Vaizey score > 12) did not differ between study groups (BTX: 7.6 + 5.3 versus SNM: 6.6 + 4.9, p = 0.07), as did the frequency of sexually active women. Serial evaluations performed after 6, 12, and 24 months post treatment showed improvement of fecal incontinence in both groups, without significant differences between them in the long-term follow-up. There were no differences between groups in the total PISQ-IR score or any of the PISQ-IR sub-scores in both sexually active and non-sexually active women after 12 months follow-up, although the proportion of sexually active women at the beginning of treatment was almost moderate (BTX: 56% and SNM: 63%, p <0.25).

The effects of BTX on intestinal function are a matter of discussion. Although more evidence is needed, therapeutic effects of BTX may be due to pelvic organ cross-sensitization, as accepted for SNM. Moreover, results from the present study demonstrate the complexity of neurophysiological interactions in the female pelvic floor and also warns about the need for a comprehensive approach to pelvic floor symptoms for a best therapeutic outcome.

## References

[1] 1. Yuaso DR, Santos JLF, Castro RA, Duarte YAO, Girão MJBC, Berghmans B, et al. Female double incontinence: prevalence, incidence, and risk factors from the SABE (Health, Wellbeing and Aging) study. Int Urogynecol J. 2018;29:265-72. 10.1007/s00192-017-3365-9 28620790

[2] 2. Amundsen CL, Komesu YM, Chermansky C, Gregory WT, Myers DL, Honeycutt EF, et al. Two-Year Outcomes of Sacral Neuromodulation Versus OnabotulinumtoxinA for Refractory Urgency Urinary Incontinence: A Randomized Trial. Eur Urol. 2018;74:66-73. 10.1016/j.eururo.2018.02.011 PMC6004242 29482936

